# Human Retrotransposon Insertion Polymorphisms Are Associated with Health and Disease via Gene Regulatory Phenotypes

**DOI:** 10.3389/fmicb.2017.01418

**Published:** 2017-08-02

**Authors:** Lu Wang, Emily T. Norris, I. K. Jordan

**Affiliations:** ^1^School of Biological Sciences, Georgia Institute of Technology, Atlanta GA, United States; ^2^PanAmerican Bioinformatics Institute Cali, Colombia; ^3^Applied Bioinformatics Laboratory, Atlanta GA, United States

**Keywords:** transposable elements, retrotransposons, Alu, L1, SVA, gene expression, gene regulation, GWAS

## Abstract

The human genome hosts several active families of transposable elements (TEs), including the Alu, LINE-1, and SVA retrotransposons that are mobilized via reverse transcription of RNA intermediates. We evaluated how insertion polymorphisms generated by human retrotransposon activity may be related to common health and disease phenotypes that have been previously interrogated through genome-wide association studies (GWAS). To address this question, we performed a genome-wide screen for retrotransposon polymorphism disease associations that are linked to TE induced gene regulatory changes. Our screen first identified polymorphic retrotransposon insertions found in linkage disequilibrium (LD) with single nucleotide polymorphisms that were previously associated with common complex diseases by GWAS. We further narrowed this set of candidate disease associated retrotransposon polymorphisms by identifying insertions that are located within tissue-specific enhancer elements. We then performed expression quantitative trait loci analysis on the remaining set of candidates in order to identify polymorphic retrotransposon insertions that are associated with gene expression changes in B-cells of the human immune system. This progressive and stringent screen yielded a list of six retrotransposon insertions as the strongest candidates for TE polymorphisms that lead to disease via enhancer-mediated changes in gene regulation. For example, we found an SVA insertion within a cell-type specific enhancer located in the second intron of the *B4GALT1* gene. *B4GALT1* encodes a glycosyltransferase that functions in the glycosylation of the Immunoglobulin G (IgG) antibody in such a way as to convert its activity from pro- to anti-inflammatory. The disruption of the *B4GALT1* enhancer by the SVA insertion is associated with down-regulation of the gene in B-cells, which would serve to keep the IgG molecule in a pro-inflammatory state. Consistent with this idea, the *B4GALT1* enhancer SVA insertion is linked to a genomic region implicated by GWAS in both inflammatory conditions and autoimmune diseases, such as systemic lupus erythematosus and Crohn’s disease. We explore this example and the other cases uncovered by our genome-wide screen in an effort to illuminate how retrotransposon insertion polymorphisms can impact human health and disease by causing changes in gene expression.

## Introduction

At least one half of the human genome sequence is derived from the replication and insertion of retrotransposons – RNA agents that transpose among chromosomal locations via the reverse transcription of RNA intermediates (Lander et al., 2001; de Koning et al., 2011). The vast majority of retrotransposon-related sequences in the human genome are derived from ancient insertion events and are no longer capable of transposition. Nevertheless, there are several families of human retrotransposons that remain active. The most abundant active families of human retrotransposons are the Alu (Batzer and Deininger, 1991; Batzer et al., 1991), LINE-1 (L1) (Kazazian et al., 1988; Brouha et al., 2003), and SVA (Ostertag et al., 2003; Wang et al., 2005) retrotransposons; recent evidence indicates that a smaller number of HERV-K endogenous retroviruses also remain capable of transposition (Wildschutte et al., 2016).

Sequences from active retrotransposon families generate insertional polymorphisms within and between human populations by means of germline transposition events. In this way, ongoing retrotranspositional activity of these RNA agents serves as an important source of human genetic variation. Retrotransposons are further distinguished by the fact that they are known to impact the regulation of human genes in a number of different ways (Feschotte, 2008; Rebollo et al., 2012; Chuong et al., 2017). Nevertheless, the joint phenotypic implications of retrotransposon generated human genetic variation, coupled with their capacity for genome regulation, have yet to be fully explored. We previously studied the implications of somatic retrotransposition for the etiology of cancer vis a vis retrotransposon induced regulatory changes in tumor suppressor genes (Clayton et al., 2016). For the current study, we were curious to understand how insertion polymorphisms generated by human retrotransposon activity may be related to commonly expressed health and disease phenotypes.

In one sense, a link between retrotransposon activity and disease is already well established. Active human retrotransposons were originally discovered due to the deleterious effects of element insertions (Kazazian et al., 1988). There are 124 genetic diseases that have been demonstrated to be caused by retrotransposon insertions, including cystic fibrosis (Alu), hemophilia A (L1) and X-linked dystonia-parkinsonism (SVA) (Hancks and Kazazian, 2012; Hancks and Kazazian, 2016). However, these cases represent so-called Mendelian diseases caused by very deleterious mutations that are expressed with high penetrance. Disease causing mutations of this kind are extremely rare and do not segregate among populations as common genetic polymorphisms. Complex multi-factorial diseases, on the other hand, are associated with more common genetic variants that exert their effects in a probabilistic as opposed to a deterministic manner. The contribution of common retrotransposon polymorphisms to complex health and disease related phenotypes has yet to be systematically explored.

Given the known connection between retrotransposon activity and genetic disease, we hypothesized that retrotransposon insertion polymorphisms may also contribute to inter-individual phenotypic differences that are associated with common diseases that have complex, multi-factorial genetic etiology. Since we previously showed that retrotransposon insertions contribute to inter-individual and population-specific differences in human gene regulation (Wang L. et al., 2016), we also hypothesized that the impact of retrotransposon insertion polymorphisms on human health could be mediated by gene regulatory effects.

Previously, it has only been possible to investigate the impact of retrotransposon polymorphisms on disease phenotypes for a limited number of individuals owing to the number of genomes that were available (Rishishwar et al., 2017). For the current study, we leveraged the accumulation of whole genome sequence and expression datasets, along with data on single nucleotide polymorphism (SNP) disease-associations, in order to perform a population level genome-wide screen for retrotransposon polymorphisms that are linked to complex health- and disease-related phenotypes.

## Materials and Methods

### Polymorphic Transposable Element (PolyTE) and SNP Genotypes

**FIGURE 1:**
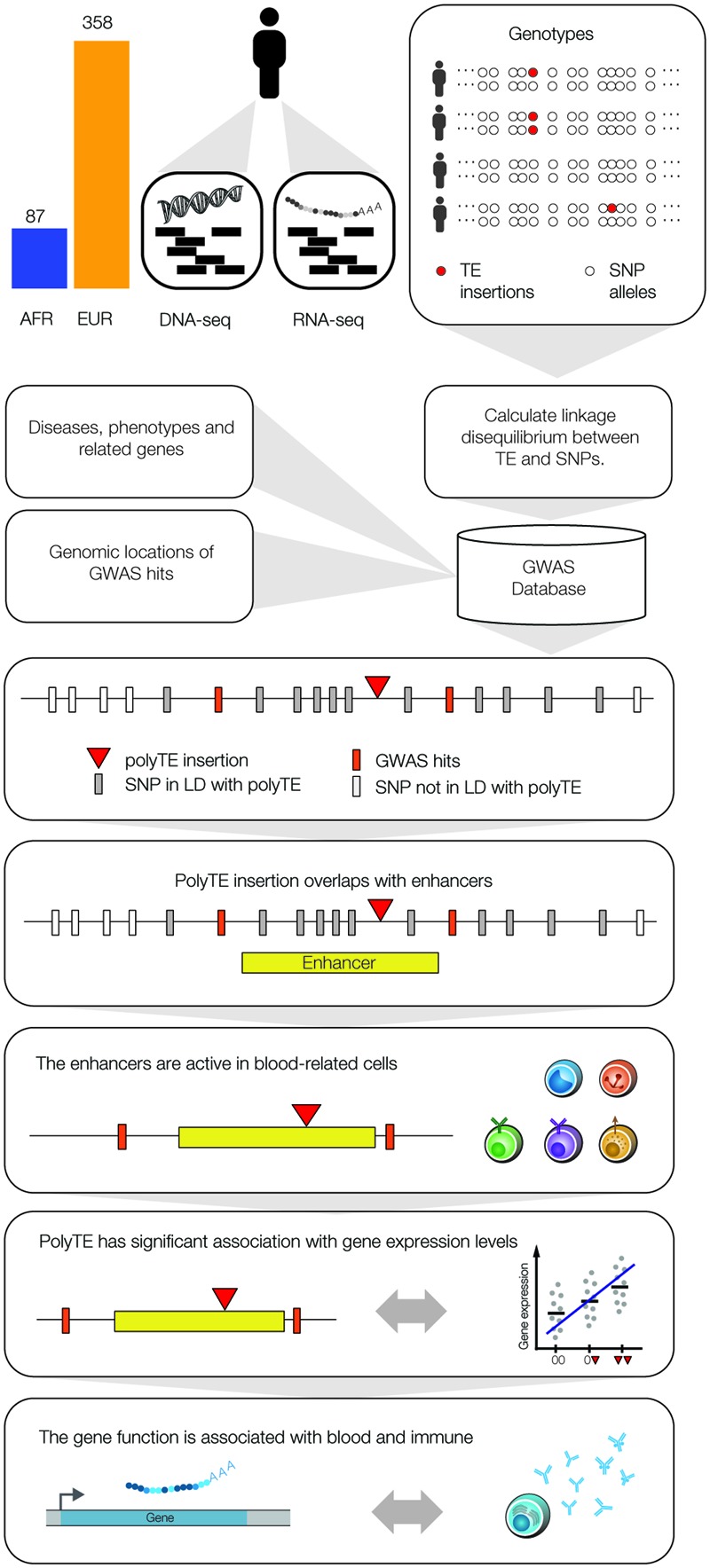
Integrative data analysis used to screen for polyTE disease-associations. Matched DNA-seq and RNA-seq samples were analyzed for 445 individuals, 87 Africans (AFR – blue) and 358 Europeans (EUR – orange). Genome-wide genotypes were characterized for polyTE insertions (presence/absence) and SNPs, and the linkage disequilibrium (LD) structure for polyTE insertions and SNP alleles was characterized for all samples. The NHGRI-EBI GWAS database was mined to extract SNP disease-associations and related information on diseases, phenotypes, genes and SNP genomic locations. A series of filters was applied to screen for a set of high-confidence polyTE disease-associations. PolyTEs were evaluated for: (1) minor allele frequency (MAF), (2) linkage to disease-associated SNPS (i.e., GWAS hits), (3) overlap with tissue-specific enhancers, (4) associations with gene expression, and (5) functional relevance for blood- and immune system-related diseases.

Human polymorphic TE (polyTE) insertion presence/absence genotypes for whole genome sequences of 445 individuals from five human populations were accessed from the phase 3 variant release of the 1000 Genomes Project (1KGP) (Altshuler et al., 2015). Whole genome SNP genotypes were taken for the same set of individuals. The phase 3 variant release corresponds to the human genome reference sequence build GRCh37/hg19, and the 5 human populations are YRI: Yoruba in Ibadan, Nigeria from Africa, CEU: Utah Residents (CEPH) with Northern and Western European Ancestry, FIN: Finnish in Finland, GBR: British in England and Scotland and TSI: Toscani in Italia from Europe. We chose these genome sequence datasets because they have matching RNA-seq data for the same individuals [see Expression Quantitative Trait Locus (eQTL) Analysis section]. The YRI population was taken to represent the African continental population group (AFR), and the four populations from Europe (CEU, FIN, GBR, and TSI) were grouped together as the European (EUR) continental population group for downstream analysis (**Figure 1**). PolyTE insertion genotypes were characterized by the 1KGP Structural Variation Group using the program MELT as previously described (Sudmant et al., 2015). Previously, we performed an independent validation the performance of this program for human polyTE insertion variant calling from whole genome sequences (Rishishwar et al., 2016). The polyTE genotype data were downloaded via the 1000 Genomes Project ftp hosted by the NCBI: http://ftp-trace.ncbi.nlm.nih.gov/1000genomes/ftp/release/20130502/ For a given polyTE insertion site in the genome, there are three possible presence/absence genotype values for an individual genome: 0-no polyTE insertion (homozygote absent), 1-a single polyTE insertion (heterozygote), and 2-two polyTE insertions (homozygote present). PolyTE genotypes were used for eQTL analysis as described in section “Expression Quantitative Trait Locus (eQTL) Analysis”. For each of the two continental population groups, only polyTE insertions and SNPs with greater than 5% minor allele frequencies (MAF) were used for the downstream analysis to ensure both the confidence of genotype calls and the reliability of the association analyses. Minor alleles for TEs are assumed to be the insertion present allele, since the ancestral state for any polyTE insertion site corresponds to the absence of an insertion (Rishishwar et al., 2015).

### PolyTE-SNP Linkage Analysis

The GCTA program (version 1.25.0) was used to estimate the linkage disequilibrium (LD) structure for polyTEs and SNPs in genomic regions centered at each polyTE insertion site. For each polyTE insertion site, pairwise correlations (*r*) between the target polyTE insertion alleles and all SNP alleles in the same LD block were computed across all individual genome samples. A correlation (*r*) significance *P*-value threshold of 0.05 was used to identify all SNPs considered to be in LD with each polyTE insertion. Pairwise distances between polyTE insertion sites and linked SNPs were calculated as the number of base pairs between each polyTE insertion site and all linked SNP locations.

### Genome Wide Association Studies (GWAS) for Disease

Associations between human genetic variants (SNPs) and health- or disease-related phenotypes were explored using the NHGRI-EBI GWAS database (MacArthur et al., 2017). GWAS database SNPs with genome-wide association values of *P* < 10^-5^ were taken for analysis, and the genomic location, specific health- or disease-related phenotype, identity of the risk allele and original reporting publications were recorded for each associated SNP. The GWAS SNPs were screened for LD with polyTE insertions as described in section “PolyTE-SNP Linkage Analysis” to yield a set of candidate disease-linked polyTE insertions for further analysis.

### Evaluating polyTE Regulatory Potential

The regulatory potential for polyTE insertions was evaluated by considering their co-location with known enhancer sequences. Active enhancers for 127 cell-types and tissues were characterized by the Roadmap Epigenomics Project using the ChromHMM program (Ernst and Kellis, 2012; Roadmap Epigenomics et al., 2015). ChromHMM integrates multiple genome-wide chromatin datasets (i.e., epigenomes), such as ChIP-seq of various histone modifications, using a multivariate Hidden Markov Model to identify the locations of tissue-specific enhancers based on their characteristic chromatin states. The data files with genomic locations for enhancers across all 127 epigenomes were accessed through the project website at http://mitra.stanford.edu/kundaje/leepc12/web_portal/chr_state_learning.html. The genomic locations of polyTE insertions that are in LD with disease-associated SNPs were compared with the genomic locations for enhancers from the 127 epigenomes, and polyTE insertions found to be located within active enhancer elements were considered to have regulatory potential. A subset of 27 epigenomes characterized for cells and tissues related to blood and the immune system – such as T cells, B cells and hematopoietic stem cells – were selected for downstream eQTL analysis [see Expression Quantitative Trait Locus (eQTL) Analysis].

The overall relative regulatory potential of polyTE insertions in a given epigenome *i* is quantified as:

$$r_{\text{i}}=\frac{t_{\text{i}}}{s_{\text{i}}}$$

where t_i_ is the proportion of polyTEs that are co-located with an enhancer element in a given epigenome *i*, and s_i_ is the proportion of SNPs from polyTE LD blocks that overlap with an enhancer element in the same epigenome *i*.

Physical associations between TE-enhancer insertions and nearby gene promoter regions were evaluated with chromatin-chromatin interaction map data, based on several different data sources, including 4C, 5C, ChIA-PET, and Hi-C, using the Chromatin Chromatin Space Interaction (CCSI) database at http://songyanglab.sysu.edu.cn/ccsi/ (Xie et al., 2016).

### Expression Quantitative Trait Locus (eQTL) Analysis

Associations between polyTE insertion genotypes and tissue-specific gene expression levels were characterized using eQTL analysis (**Figure 1**). PolyTE insertion presence/absence genotypes were characterized as described in section “Polymorphic Transposable Element (polyTE) and SNP Genotypes”. RNA-seq gene expression data for the same 445 individual genome samples used for polyTE genotype characterization were taken from the GEUVADIS RNA sequencing project. Genome-wide expression levels were measured for the same lymphoblastoid cell lines, i.e., Epstein–Barr virus (EBV) transformed B-lymphocytes (B cells), as used for DNA-seq analysis in the 1KGP. RNA isolation, library preparation, sequencing and read-to-genome mapping were performed as previously described (Lappalainen et al., 2013). As with the polyTE genotype data, the RNA-seq reads were mapped to the human genome build GRCh37/hg19. The process of gene expression normalization and quantification based on these RNA-seq data has been extensively validated as part of the GEUVEDIS project (t Hoen et al., 2013). The GEUVADIS RNA-seq data were used to compute gene expression levels for ENSEMBL gene models as previously described (Flicek et al., 2013). Normalization of gene expression levels was done using a combination of a modified reads per kilobase per million mapped reads (RPKM) approach followed by the probabilistic estimation of expression residuals (PEER) method as previously described (Stegle et al., 2012). This procedure has been shown to eliminate batch effects among different RNA-seq samples and to reduce the overall variance across samples, thereby ensuring the most accurate and comparable gene expression level inferences among samples. The normalized gene expression levels were accessed from the GUEVADIS project ftp server hosted at the EBI: ftp://ftp.ebi.ac.uk/pub/databases/microarray/data/experiment/GEUV/E-GEUV-1/analysis_results/.

PolyTE insertions that are (1) linked to at least one disease-associated SNP, and (2) located within a blood- or immune system-related enhancer were taken as a candidate set for eQTL analysis with the lymphoblastoid cell line RNA-seq data. PolyTE insertion presence/absence genotypes were regressed against gene expression levels to identify eQTLs (TE-eQTLs) using the program Matrix eQTL (Shabalin, 2012). Matrix eQTL was run using the additive linear (least squares model) option with gender and population used as covariates. This was done for all possible pairs of polyTE insertion sites from the candidate set and all genes. *Cis* vs. *trans* TE-eQTLs were defined later as polyTE insertion sites that fall inside (*cis*) or outside (*trans*) 1 megabase from gene boundaries. *P*-values were calculated for all TE-eQTL associations, and FDR *q*-values were then calculated to correct for multiple statistical tests. The genome-wide significant TE-eQTL association threshold was set at FDR *q* < 0.05, corresponding to *P* = 4.7 × 10^-7^ (AFR) and *P* = 2.6 × 10^-7^ (EUR).

### Interrogation of Disease-Associated Gene Function and Association Consistency

The potential functional impacts of disease-associated TE-eQTL were evaluated via comparison of annotated gene functions and reported GWAS phenotypes for polyTE-linked SNPs. Gene functions were taken from the NCBI Entrez gene summaries, and GWAS phenotypes were taken from the original literature where the associations were reported. Genes that were found to be functionally related to GWAS reported health- or disease-related phenotypes were further checked for the direction of association. If the GWAS SNP-gene pair shows the same direction of association as the polyTE-gene pair, then the pair was included in the final set of significant gene-polyTEs association pairs (**Table 1**). For each gene in the final set, its tissue-specific expression levels across 18 tissues, including 4 blood- and immune-related tissues, were taken from the Illumina BodyMap and GTEx projects (Flicek et al., 2013; Mele et al., 2015; Rivas et al., 2015).

**Table 1:** PolyTE disease associations.

PolyTE^a^	Chr^b^	Pos^b^	GWAS SNP^c^	GWAS Phenotype^c^	GWAS gene^c^	GWAS *P*-value^c^	#Enhancer Overlaps^d^	eGene^d^	eQTL *P*-value^d^	eQTL Type^d^
Alu-2829	3	154966214	rs13064954	Diabetic retinopathy	*LINC00881, CCNL1*	7.00E-07	1	*LILRA1*	5.94e-10	*Trans*
Alu-5072	6	32589834	rs4530903	Lymphoma	*TRNAI25*	2.00E-08	20	*HLA-DRB5*	8.49e-13	*Cis*
Alu-5075	6	32657952	rs2858870	Nodular sclerosis Hodgkin lymphoma	*TRNAI25*	8.00E-18	15	*HLA-DQB1-AS1*	1.36e-11	*Cis*
SVA-282	6	33030313	rs3077	Chronic hepatitis B infection	*HLA-DPA1*	5.00E-39	6	*HLA-DPB2*	1.05e-13	*Cis*
SVA-401	9	33130564	rs10758189	IgG glycosylation	*B4GALT1*	2.00E-06	4	*B4GALT1*	4.47e-20	*Cis*
SVA-438	10	17712792	rs6602203	Glucose homeostasis traits	*ST8SIA6-AS1, PRPF38AP2*	5.00E-06	7	*TMEM236*	1.30e-07	*Cis*

*^a^PolyTE insertion identifier following the nomenclature of the 1KGP structural variation group. ^b^Genomic location of the polyTE. ^c^Information on the linked disease-associated GWAS SNP, disease phenotype and gene. ^d^Information on the regulatory potential of the polyTE insertion based on co-location with enhancers and eQTL analysis.*

## Results

**FIGURE 2:**
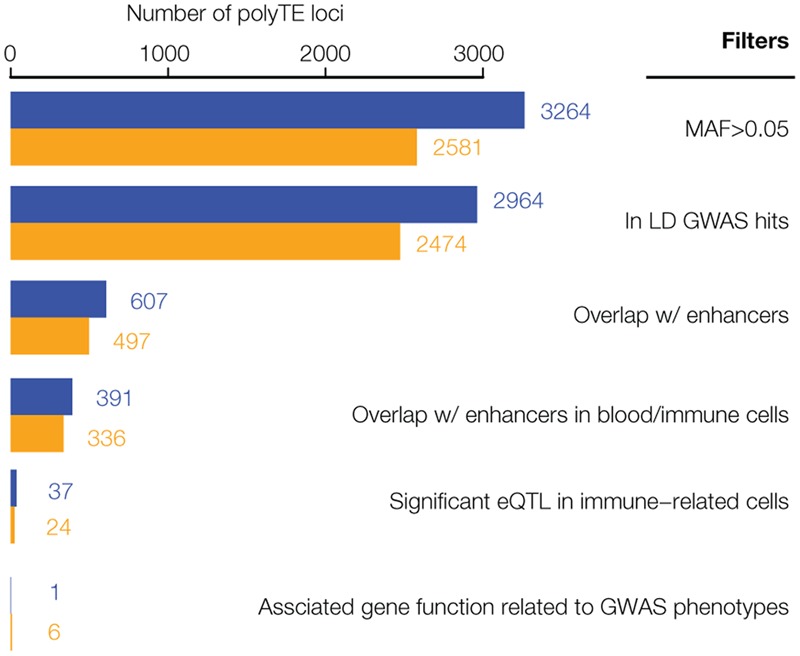
Results of the genome-wide screen for polyTE disease-associations. As illustrated in **Figure 1**, a series of filters was applied to screen for a final set of high-confidence polyTE disease-associations. The number of polyTE insertions that remain after the application of each successive filter is shown for the African (AFR – blue) and European (EUR – orange) population groups.

We used a genome-scale data analysis approach to explore the potential impact of human genetic variation generated by the activity of TEs on health and disease (**Figure 1**). This approach entailed an integrative analysis of (1) TE insertion polymorphisms, (2) SNPs, (3) SNP-disease associations, (4) tissue-specific enhancers, (5) eQTL, and (6) gene function/expression profiles. The rationale behind this approach was to employ a series of successive genome-wide filters, which would converge on a set of high-confidence TE insertion polymorphisms that are most likely to impact health- or disease-related phenotypes. Our analysis started with 5,845 polyTE insertions, with MAF > 0.05 for two continental population groups (European and African), and converged on a final set of seven high-confidence TE disease-association candidates (**Figure 2**). The final set of seven health/disease-implicated TE insertion polymorphisms that we found are distinguished by their linkage to disease-associated SNPs as well as their regulatory and functional properties. We describe the results and implications for each step in our TE disease-association screen in the sections below.

### Linkage Disequilibrium for PolyTEs and Disease-Associated SNPs

The genomic locations of polyTE insertions were characterized for 445 individuals from one African (AFR) and four European (EUR) populations as described in section “Polymorphic Transposable Element (polyTE) and SNP Genotypes” of the Materials and Methods. This was done for the most common families of active human TEs: Alu, L1, and SVA. For each polyTE insertion location, individual genotypes were characterized as homozygous absent (0), heterozygous (1), or homozygous present (2). The distributions of polyTE insertion genotypes among individuals from each population were used to screen for polyTEs that are found at relatively high MAF > 0.05. The LD structure of the resulting common polyTE insertions, with adjacent common SNPs (also at MAF > 0.05), was then defined using correlation analysis across individual genome samples (see Materials and Methods section PolyTE-SNP Linkage Analysis). In addition, the genomic locations of common polyTE insertion variants and their linked SNPs were compared to the locations of disease-associated SNPs reported in the NHGRI-EBI GWAS database [see Materials and Methods Genome Wide Association Studies (GWAS) for Disease]. Linkage correlation coefficients between all polyTE insertions analyzed here and GWAS SNPs are shown in **Supplementary Table S1**.

**FIGURE 3:**
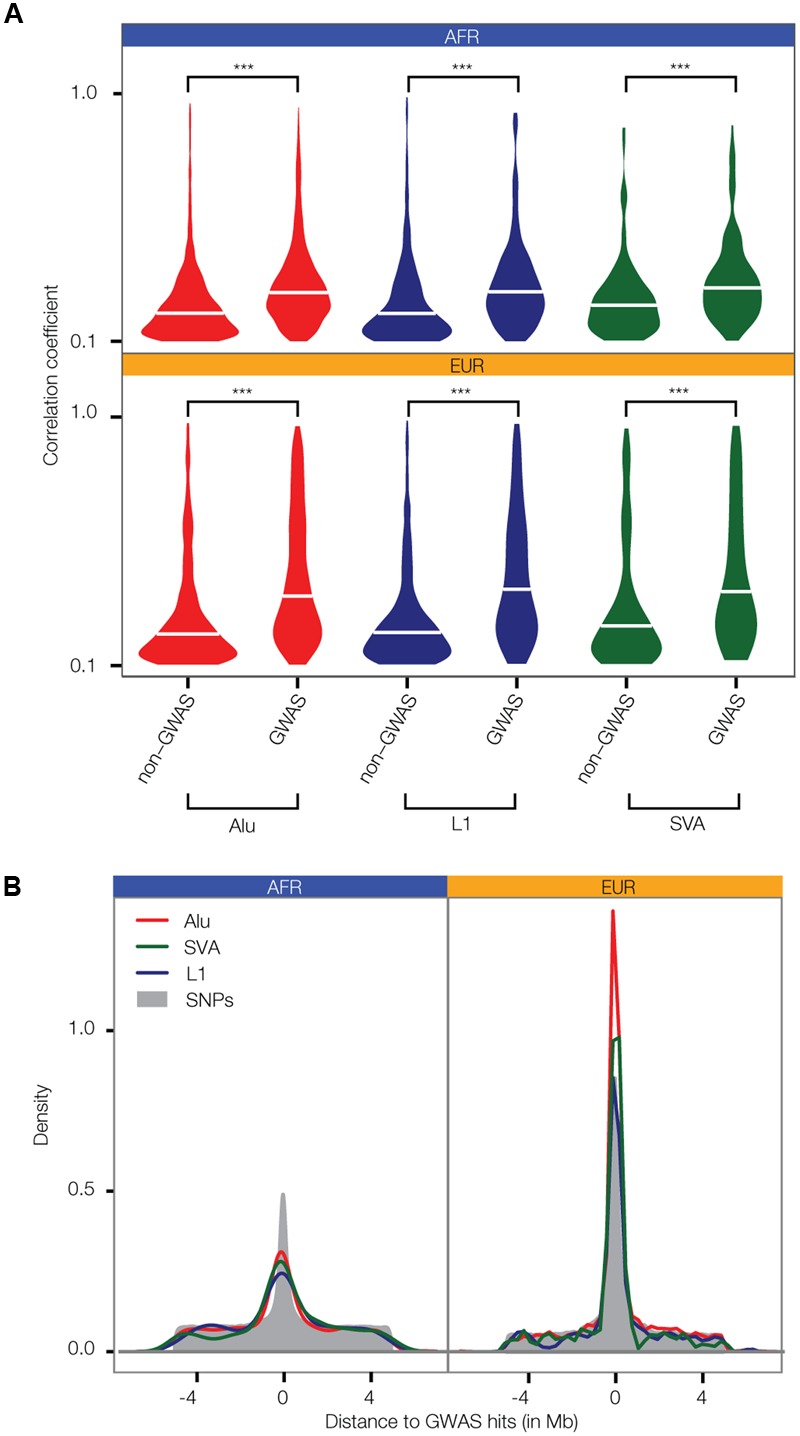
Linkage between polyTE insertions and SNP disease-associations from GWAS. **(A)** Distributions of the allele correlation coefficients (*r*) are shown for (1) polyTE insertions and non-GWAS SNPs, and (2) polyTE insertions and GWAS SNPs. Higher correlation coefficients indicate tighter linkage. The significance of the differences for the non-GWAS SNP vs. the GWAS SNP correlation coefficient distributions are indicated (Kolmogorov–Smirnov test; ^∗∗∗^*P* < 2.4 × 10^-5^). **(B)** Density distributions of the distance between polyTE insertions and SNPs to the nearest GWAS disease-associated SNP. Correlation coefficient **(A)** and density **(B)** distributions are shown separately for the Alu (red), L1 (blue), and SVA (green) TE families in the African (AFR – blue) and European (EUR – orange) population groups.

Distributions of LD correlations between polyTEs and adjacent SNPs were compared separately for non-disease-associated vs. disease-associated SNPs. For all three families of active human TEs, in both the AFR and EUR population groups, polyTEs are found in significantly higher LD with disease-associated SNPs compared to non-disease-associated SNPs (**Figure 3A**). In addition, polyTE variants are located closer to disease-associated SNPs than non-disease-associated SNPs for the EUR population group (**Figure 3B**). A similar enrichment was not seen for the AFR population group, which may be attributed to the lower number of samples available for analysis for this group (AFR = 87 vs. EUR = 358). Indeed, when a larger number of AFR samples, which do not have matched RNA-seq data, were used for the same linkage analysis, the results were qualitatively identical to those seen for the EUR samples analyzed here. Taken together, these results indicate that polyTEs are more likely to be tightly linked to disease-associated SNPs compared to adjacent linked SNPs from the same LD blocks, suggesting a possible role in disease etiology for some TE variants. This is notable in light of the facts that (1) the TE genotypes were not considered in the initial association studies, and (2) TE insertions entail substantially larger-scale genetic variants than SNPs. Thus, polyTEs found on the same haplotypes as disease-associated SNPs may be expected to have an even greater impact on health- and disease-outcomes in some cases.

### Co-location of Disease-Linked PolyTEs with Tissue-Specific Enhancers

**FIGURE 4:**
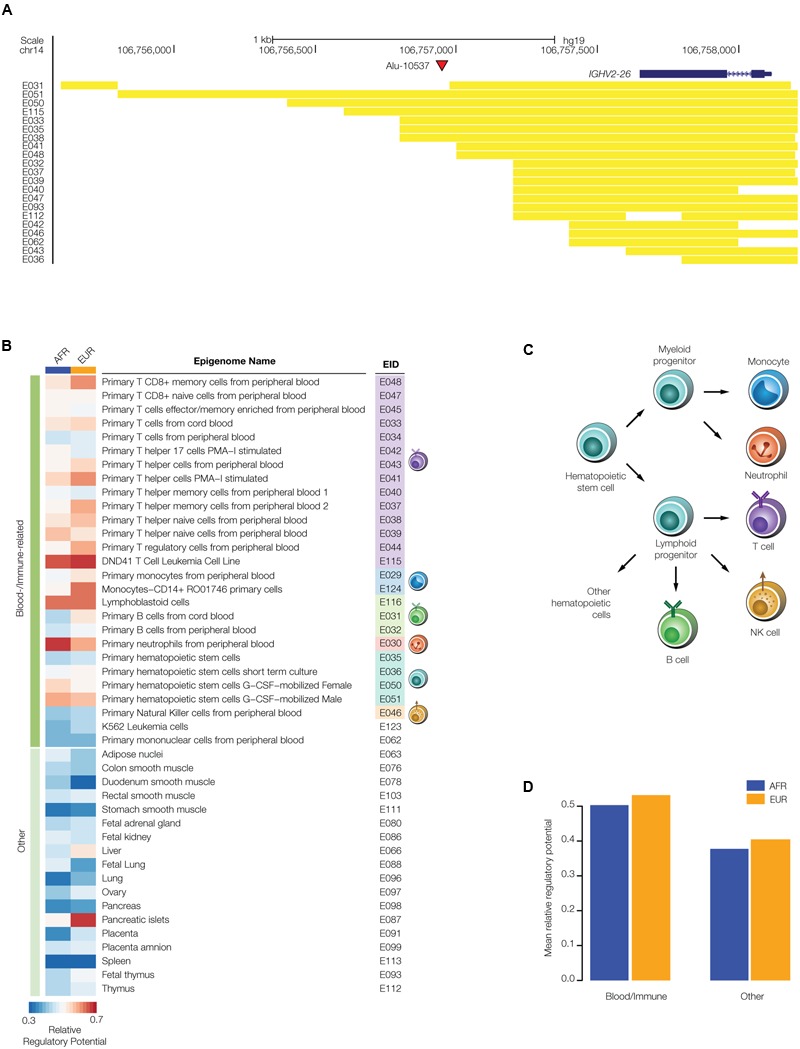
Regulatory potential of disease-linked polyTE insertions. PolyTE insertions linked to disease-associated SNPs were evaluated for their co-location with tissue-specific enhancers. **(A)** UCSC Genome Browser screen capture showing an example of a polyTE insertion – Alu-10537 – that overlaps with a number of tissue specific enhancers. The genomic location of the Alu insertion on chromosome 14, downstream of the *IGHV2-26* gene (blue gene model), is indicated with a red arrow. The genomic locations of co-located enhancers, characterized based on chromatin signatures from a variety of tissue-specific epigenomes, are indicated with yellow bars. **(B)** Heatmap showing the relative regulatory potential (see Materials and Methods section Evaluating polyTE Regulatory Potential) of polyTE insertions for a variety of tissue-specific epigenomes. Blood- and immune-related tissues are shown separately from examples of the other tissue types analyzed here. **(C)** Developmental lineage of immune-related cells for which enhancer genomic locations were characterized. **(D)** The mean relative regulatory potential for disease-linked polyTE insertions is shown for blood- and immune-related tissues compared to all other tissue-types analyzed here. Values for the African (AFR – blue) and European (EUR – orange) population groups are shown separately.

Given the fact that TEs are known to participate in human gene regulation via a wide variety of mechanisms (Feschotte, 2008; Rebollo et al., 2012; Chuong et al., 2017), we hypothesized that polyTEs may impact disease by virtue of gene regulatory effects. The regulatory potential of polyTEs linked to disease-associated SNPs was first evaluated by searching for insertions that are co-located with tissue-specific enhancers. The locations of enhancers were characterized for 127 cell- and tissue-types based on their chromatin signatures as described in section “Evaluating polyTE Regulatory Potential” of the Materials and Methods. An example of an enhancer co-located with a disease-linked polyTE is shown for an Alu element that is inserted 5′ to the Immunoglobulin Heavy Variable 2-26 (*IGHV2-26*) encoding gene (**Figure 4A**). We found a total 607 disease-linked polyTEs co-located with enhancers in the AFR population group and 437 in EUR group; 391 (AFR) and 336 (EUR) of those enhancers correspond to blood- or immune-related tissues (**Figure 2**). Details on the co-localization of polyTE insertions and the enhancers characterized for each epigenome are shown in **Supplementary Table S2**.

We estimated the overall regulatory potential for disease-linked polyTEs in all cell- and tissue-types by computing the relative ratio of enhancer co-located insertions as described in section “Evaluating polyTE Regulatory Potential” of the Materials and Methods. The results of this analysis are considered separately for the blood- and immune-related tissues (**Figure 4B**) and all other tissues from which enhancers were characterized. Enhancer co-located disease-linked polyTEs from blood/immune cell-types show higher overall regulatory potential than ones that are co-located with enhancers characterized for the other tissue-types (**Figures 4C,D**). These results suggest that the set of disease-linked polyTEs studied here may have a disproportionate impact on immune-related diseases, and we focused our subsequent efforts on this subset of health conditions.

### Expression Associations for Disease-Linked and Enhancer Co-located PolyTEs

**FIGURE 5:**
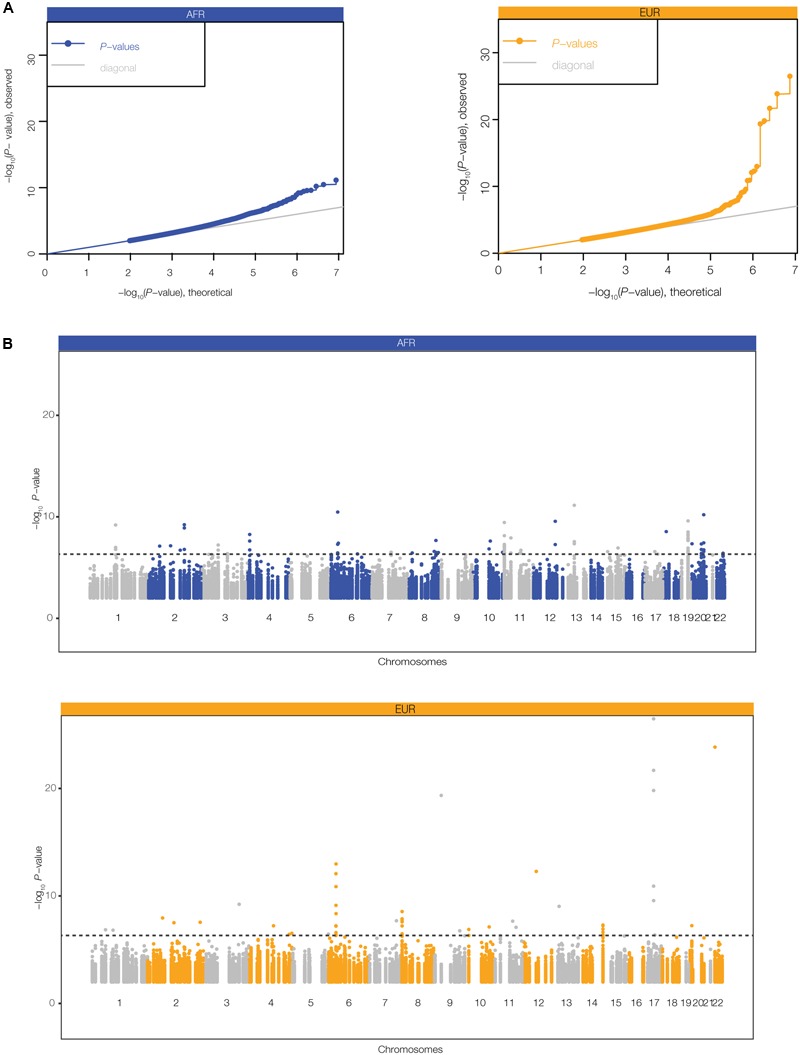
Expression quantitative trait (eQTL) analysis for disease-linked polyTEs. eQTL analysis was performed by regressing lymphoblastoid gene expression levels against polyTE insertion genotypes for the for the African (AFR – blue) and European (EUR – orange) individuals analyzed here. **(A)** Quantile–quantile (Q–Q) plots relating the observed (*y*-axis) to the expected (*x*-axis) TE-eQTL log transformed *P*-values. **(B)** Manhattan plots showing the genomic distributions of TE-eQTL log transformed *P*-values. The dashed line corresponds to a false discovery rate (FDR) threshold of *q* < 0.05, corresponding to *P* = 4.7 × 10^-7^ (AFR) and *P* = 2.6 × 10^-7^ (EUR).

We further evaluated the regulatory potential of the polyTEs that were found to be both disease-linked and co-located with blood- and immune-related enhancers using an eQTL approach [see Materials and Methods section Expression Quantitative Trait Locus (eQTL) analysis]. Genotypes for this subset of polyTEs from the 445 genome samples analyzed here were regressed against gene expression levels characterized from lymphoblastoid cell lines for the same individuals. Quantile–quantile (Q–Q) plots comparing the observed vs. expected *P*-values for the e-QTL analysis in the AFR and EUR population groups are shown in **Figure 5A**, revealing a number of statistically significant associations that are likely to be true-positives. There are 83 (AFR) and 42 (EUR) genome-wide significant TE-eQTL (**Supplementary Table S3**), and they are enriched in genomic regions that encode immune-related genes (**Figure 5B**). We narrowed this list further by selecting the strongest TE-eQTL association for each individual polyTE, resulting in a final list of 37 (AFR) and 24 (EUR) immune-related TE-eQTL (**Figure 2**).

**FIGURE 6:**
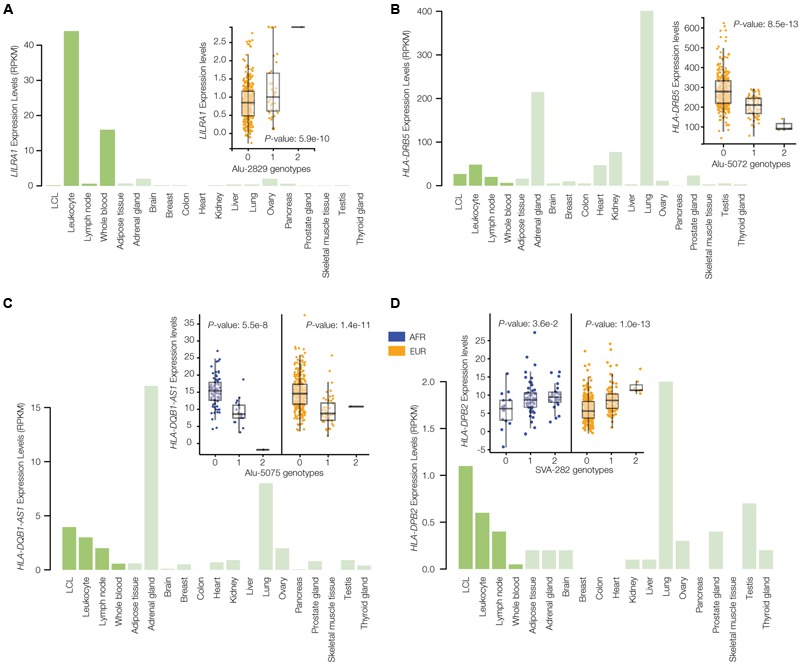
Gene expression profiles and eQTL results for disease-associated polyTE insertions. Bar-charts of tissue-specific expression levels and box-pots of eQTL analyses are shown for four examples of TE-eQTLs corresponding to disease-linked and enhancer co-located polyTE insertions that regulate immune-related genes: **(A)** Alu-2829 and *LILRA1*, **(B)** Alu-5072 and *HLA-DRB5*, **(C)** Alu-5075 and *HLA-DQB1-AS1*, and **(D)** SVA-282 and *HLA-DPB2*. Bar-charts show tissue-specific expression levels as normalized RPKM values (green). The inset eQTL box-plots show individual sample gene expression levels (*y*-axis) regressed against polyTE insertion presence/absence genotypes (*x*-axis): 0-homozygous absent, 1-heterozygous, 2-homozygous present. Each dot represents a single individual from the African (AFR – blue) and/or European (EUR – orange) population groups.

The results of the TE-eQTL analysis further underscore the regulatory potential of the disease-linked polyTEs characterized here and also allowed us to narrow down the list of candidate insertions. Starting with the list of TE-eQTL, we searched for ‘consistent’ examples where the disease-linked polyTE is associated with the expression of a gene that is functionally related to the annotated disease phenotype. This allowed us to converge on a final set of seven high-confidence disease-associated TE insertion polymorphisms (**Figure 2** and **Table 1**). Four of these disease-associated polyTEs are illustrated in **Figure 6**, and we provide additional information on two examples in the following section “Effects of polyTE Insertions on Immune- and Blood-Related Conditions.” The four examples shown in **Figure 6** all correspond to polyTEs that are linked to disease-associated SNPs and co-located with enhancers characterized from blood- or immune system-related tissues; in addition, the genes that these polyTE insertions regulate are all known to function in the immune system.

**FIGURE 7:**
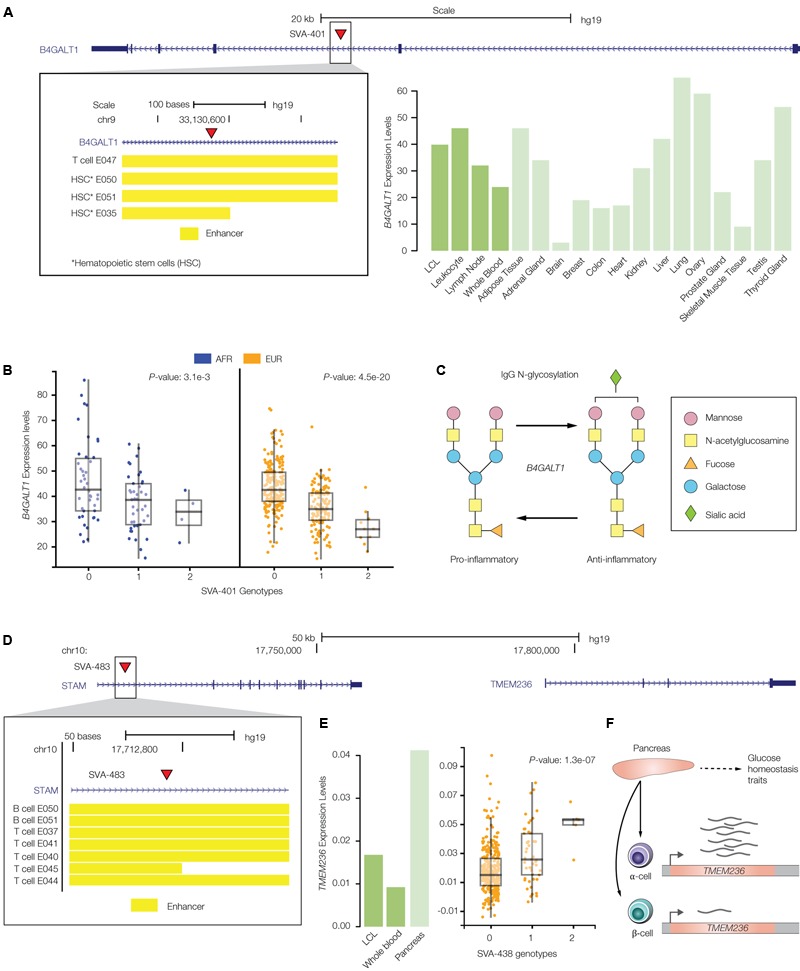
PolyTE insertions associated with immune- and blood-related conditions. **(A)** UCSC Genome Browser screen capture showing the location of the SVA-401 insertion (red arrow) on chromosome 19 within the second exon of the *B4GALT1* gene. The inset shows the genomic locations of co-located enhancers, characterized based on chromatin signatures from a variety of tissue-specific epigenomes locations, as yellow bars. The bar-chart shows *B4GALT1* tissue-specific expression levels as normalized RPKM values (green). **(B)** eQTL box-plots show individual sample gene expression levels (*y*-axis) regressed against SVA-401 insertion presence/absence genotypes (*x*-axis): 0-homozygous absent, 1-heterozygous, 2-homozygous present. Each dot represents a single individual from the African (AFR – blue) or European (EUR – orange) population groups. **(C)** *B4GALT1* catalyzed glycosylation of the Immunoglobulin G (IgG) antibody, resulting in conversion from pro- to anti-inflammatory activity. **(D)** UCSC Genome Browser screen capture showing the location of the SVA-438 insertion (red arrow) on chromosome 10 within the first exon of the *STAM* gene, upstream of the regulated *TMEM236* gene. The inset shows the genomic locations of co-located enhancers (yellow bars). **(E)** Bar-chart of *TMEM236* tissue-specific expression levels and box-pot of the SVA-438 *TMEM236* eQTL analyses. **(F)** Functional role and cell-type specific expression profile for *TMEM236*.

Six of the seven disease-associated polyTE insertions are considered to be population-specific, based on significant eQTL results in only one population, whereas a single case is shared between both the AFR and EUR population groups (**Figure 6C**). However, two of the six cases considered to be population-specific using the eQTL criterion do show consistent trends across populations but failed to reach genome-wide significance when controls for multiple statistical tests were implemented (**Figures 6D, 7B**).

### Effects of PolyTE Insertions on Immune- and Blood-Related Conditions

Here, we described two specific examples of the effects that polyTE insertions can exert on immune- and blood-related disease phenotypes. **Figure 7A** shows the SVA-401 insertion that is co-located with a cell-type specific enhancer found in the second intron of the Beta-1,4-Galactosyltransferase 1 (*B4GALT1*) encoding gene, which is normally expressed at high levels in immune-related tissues. Chromatin interaction maps characterized for several different cell types – CD34, GM12878, and Mcf7 – show that this *B4GALT1* intronic enhancer physically associates with the gene’s promoter region. The disruption of the *B4GALT1* enhancer by the SVA insertion is associated with down-regulation of the gene in B-cells, for both AFR and EUR population groups (**Figure 7B**). *B4GALT1* encodes a glycosyltransferase that functions in the glycosylation of the Immunoglobulin G (IgG) antibody in such a way as to convert its activity from pro- to anti-inflammatory (**Figure 7C**) (Kaneko et al., 2006; Anthony and Ravetch, 2010; Lauc et al., 2013). Down-regulation of this gene in individuals with the enhancer SVA insertion should thereby serve to keep the IgG molecule in a pro-inflammatory state. Consistent with this idea, the *B4GALT1* enhancer SVA insertion is linked to a genomic region implicated by GWAS in both inflammatory conditions and autoimmune diseases such as systemic lupus erythematosus and Crohn’s disease (Lauc et al., 2013).

Another example of an SVA insertion into an enhancer element is shown for the adjacently located Signal Transducing Adaptor Molecule (*STAM*) and Transmembrane Protein 236 (*TMEM236*) encoding genes. The SVA-438 insertion is co-located with an enhancer in the first intron of the *STAM* gene (**Figure 7D**), but its presence is associated with changes in expression of the nearby *TMEM236* gene (**Figure 7E**). *TMEM236* is located ∼100 kbp downstream of the SVA-438 insertion and is most highly expressed in pancreatic islet α-cells (**Figure 7F**) (Ackermann et al., 2016; Wang Y.J. et al., 2016). Islet α-cells function to secrete glucagon, a peptide hormone that elevates glucose levels in the blood (Quesada et al., 2008). The SVA-438 insertion is associated with increased expression of *TMEM236*, which would be expected to lead to increased blood glucose levels. This expectation is consistent with the fact that the SVA-438 insertion is also linked to the risk allele (T) of the SNP rs6602203, which is associated with a reduced metabolic clearance rate of insulin (MCRI), an endophenotype that is associated with the risk of type 2 diabetes (Palmer et al., 2015). In other words, up-regulation of *TMEM236* by the SVA-438 insertion may be mechanistically linked to insulin resistance by virtue of increasing blood sugar and decreasing insulin clearance.

## Discussion

The results reported here underscore the influence that retrotransposon insertion polymorphisms can exert on human health- and disease-related phenotypes. The integrative data analysis approach that we took for this study also revealed how polyTE disease-associations are mediated by the gene regulatory properties of retrotransposon insertions. We adopted a conservative approach to screen for the potential regulatory effects of retrotransposon insertions by choosing candidate elements as those that were inserted into regions previously defined as tissue-specific enhancers in blood/immune cells. Retrotransposons that insert into enhancer sequences could entail loss-of-function mutants by virtue of disrupting enhancer sequences, or they could serve as gain-of-function mutants by altering enhancer activity. Our results can be considered to show instances of both loss- and gain-of-function enhancer mutations with respect to the decrease or increase, respectively, of gene expression levels that are associated with element insertion genotypes (**Figures 6, 7**). Nevertheless, it is worth noting that our conservative approach could be prone to false negatives as it would not uncover novel enhancer activity provided by element insertions at new locations in the genome.

The TE regulatory findings that we report here are consistent with previous studies showing that TE-derived sequences have contributed a wide variety of gene regulatory elements to the human genome (Feschotte, 2008; Rebollo et al., 2012; Chuong et al., 2017), including promoters (Jordan et al., 2003; Marino-Ramirez et al., 2005; Conley et al., 2008), enhancers (Bejerano et al., 2006; Kunarso et al., 2010; Chuong et al., 2013, 2016; Notwell et al., 2015), transcription terminators (Conley and Jordan, 2012) and several classes of small RNAs (Weber, 2006; Piriyapongsa et al., 2007; Kapusta et al., 2013). Human TEs can also influence gene regulation by modulating various aspects of chromatin structure throughout the genome (Lander et al., 2001; Pavlicek et al., 2001; Schmidt et al., 2012; Jacques et al., 2013; Sundaram et al., 2014; Wang et al., 2015).

It is important to note that the research efforts which have uncovered the regulatory properties of human TEs, including a number of our own studies, have dealt exclusively with sequences derived from relatively ancient insertion events. These ancient TE insertions are present at the same (fixed) locations in the genome sequences of all human individuals. In other words, previously described TE-derived regulatory sequences are uniformly present among individual human genomes and thereby do not represent a source of structural genetic variation. Such fixed TE-derived regulatory sequences may not be expected to provide for gene regulatory variation among individuals or for that matter to contribute to inter-individual differences related to health and disease.

Nevertheless, we recently showed that TE insertion polymorphisms also exert regulatory effects on the human genome (Wang L. et al., 2016). Specifically, polyTE insertions were shown to contribute to both inter-individual and population-specific differences in gene expression and to facilitate the re-wiring of transcriptional networks. The results reported here extend those findings up the hierarchy of human biological organization by revealing potential mechanistic links between polyTE-induced gene regulatory changes and the endophenotypes that underlie human health and disease.

## Author Contributions

LW performed all of the analyses described in the study. EN contributed all GWAS data used in the study. IJ conceived of, designed and supervised the study. All authors contributed to the drafting and revision of the manuscript.

## Conflict of Interest Statement

The authors declare that the research was conducted in the absence of any commercial or financial relationships that could be construed as a potential conflict of interest.

## Supplementary Material

TABLE S1 Linkage disequilibrium (LD) structure between polyTEs and GWAS disease-associated SNPs.

TABLE S2 Genomic co-localization of polyTEs with tissue-specific enhancers.

TABLE S3 Expression quantitative trait loci (eQTL) results for disease-linked and enhancer co-located polyTEs.
